# Research in Reproduction: Challenges, Needs, and Opportunities

**DOI:** 10.3389/fphys.2017.00046

**Published:** 2017-02-08

**Authors:** Richard Ivell

**Affiliations:** ^1^School of Biosciences and School of Veterinary Medicine and Science, University of NottinghamNottingham, UK; ^2^School of Biological Sciences, University of AdelaideAdelaide, SA, Australia

**Keywords:** neohormone, systems biology, endocrine noise, uterine peristalsis, DOHaD, spermatozoon, environmental disrupting chemicals, livestock reproduction

After delivering a student lecture on the quantitative attributes of human infertility, the very poor quality of human sperm in comparison to other species, and the biologically unlikely long “time-to-pregnancy” statistics even among young people, I generally pose to my audience of several hundred young and healthy students the following question. “All of these characteristics would be enough to make most wild species go extinct; look around you; is that what is happening to us? If not, why not?” Fairly quickly, they begin to talk about medical information and research, IVF (*in vitro* fertilization), etc. This situation hides, however, an important paradox for those who work in or fund research in reproduction. Namely, it is one thing to look at the level of the population, and quite another to look at the individual. Many not directly involved tend to see the former level, with overpopulation an issue, and contraception mostly a socio-economic matter, and anyway infertility is not life-threatening, like cancer. For the affected individual or couple, it looks very different. Moreover, we are confronted with severe and unresolved issues in regard to livestock reproduction, as well as for endangered wildlife.

In the following, selected research challenges in reproduction are listed. This is not exhaustive and less about their clinical importance, but more about the extent of our knowledge deficit and the potential translational impact such basic research could have. It will be noticeable that in many of these areas a paradigm shift in our thinking may be required. So much of current thinking about research in reproduction still relies on simplistic concepts that are at least 50 years old. The reproductive system is different from most other physiological systems in the body in that its functionality in the adult depends on a highly dynamic but very tightly controlled proliferation and differentiation of specialized cell types. And while deficits in functionality may not necessarily be life-threatening, its close informational interdependence with other physiological systems nevertheless gives it great importance in our understanding of health and disease.

## Neohormones and evolution

The vast majority of interventions for fertility treatment or contraception refer to only a small number of hormones involved with the hypothalamo-pituitary-gonadal (HPG) axis, namely gonadotropins, GnRH (gonadotropin releasing hormone), and the sex steroids, all of which were identified many decades ago. Whilst these are without doubt central to the control of reproduction in both males and females, since then many hundreds of new regulatory molecules have been identified, but few if any have been applied in a clinical context to regulate reproduction. Of these novel molecules, the neohormones are of particular interest. These are defined as hormone-receptor systems which evolved concurrently with the emergence of mammals from their reptilian ancestors to address the specifically mammalian defining traits of viviparity, internal fertilization, maternal adaptation to pregnancy, lactation, and reproductive behavior (Anand-Ivell et al., 2013). Besides human chorionic gonadotropin (hCG) in humans, interferon-tau in ruminants, and oxytocin, the relaxin family of insulin-like peptides have received most attention. However, except for hCG and oxytocin, none of these have entered significantly into the treatment of reproductive conditions in any species, even though relaxin itself has been shown to be very effective in reducing pregnancy loss in monkeys (Goldsmith et al., 2004; Einspanier et al., 2009). One part of the problem is that gaining regulatory approval for novel agents in the context of reproduction and potential pregnancy is very difficult; another is the simple cost of developing appropriate compounds whose synthesis is currently very expensive, though some new non-peptide mimetics appear to be on the horizon (Agoulnik et al., 2016). The result is that currently the majority of pharmaceutical interventions in reproduction are based on science which is at least 50 years old. This situation needs to change.

## Systems approaches vs. the magic bullet—the myth of the golden biomarker

Historically, research in reproduction, as in most areas of physiology, has been dominated by the notion that there can be a single biomarker reflecting a particular health status, and as a corollary a single molecular target which can pharmaceutically resolve a health issue. For many aspects of reproduction, from oocyte and blastocyst status, to implantation and early pregnancy loss, this goal is still a long way off, with many modern global approaches in transcriptomics, epigenomics, and proteomics failing to identify an unambiguous and reproducible signature for a particular condition. Part of the issue may be in our rudimentary bioinformatic ability to identify an appropriate “fingerprint” amongst the huge amount of data now available. But part may also be in our still rather static paradigm of what is health and what is disease (see below).

## Endocrine “noise” and implantation

We now recognize that in any dialogue between component cells and tissues a multiplex of diverse factors (hormones, metabolites), their receptors, and signaling pathways are involved, to form a dynamic and complex network of interactions. In differentiation, in health and disease, there are subtle shifts in the coordinates of such a network, which may or may not be reflected in individual parameters. This is why, for example, there are so many instances of gene deletions in mice (a very gross intervention) with no obvious phenotypic effect, or with an impact only under stress conditions, or with other concomitant deletions. Hence when we use the term “redundancy” for a particular gene product, this does not imply that it is without function, but merely that we do not yet understand its physiological context. Evolution has honed the genomes of all extant species to represent the perfect compromise for that organism in its ecological context.

What is becoming clear, and what we still cannot really grasp in a scientific sense, is the dynamic nature of any particular informational network. This leads to the notion of “noise” in a system. For example, in the developing oocyte or early embryo, health may be associated with a “quiet” system, with low statistical variance of its component interactions (Leese, 2012). At implantation, there is a dialogue between the implanting blastocyst and the endometrium, involving multiple signaling pathways. In a healthy situation such informational exchange will be minimal, but in an unhealthy situation the dialogue between embryo and endometrium will intensify; there will be an increase in endocrine “noise.” This may resolve itself and implantation succeeds, or it may worsen and lead to rejection (Macklon and Brosens, 2014). The key point to note here is that measurement of a single element in that conversational network may only show an increase in variance, i.e., noise, without any impact on average parameter values.

To extend this concept further, it has been shown that the impact of some endocrine disrupting chemicals (EDCs) may be to induce a significant increase in parameter variance without affecting the parameter mean. Such results are also linked to the concept of responders vs. non-responders, which can statistically mask real physiological effects (Bellingham et al., 2012).

Thus, one of the major challenges facing research in reproductive physiology will be to develop and quantify ways of assessing the dynamics of intercellular networks, such as to measure “noise” and treat this as a parameter in its own right, with variance being given as much importance as the mean.

## Uterine peristalsis and disease

As an organ, the uterus is a highly dynamic structure with its functional endometrium enclosed within a regulated muscular envelope, the myometrium. It is thus much like the gut, though surprisingly we know far less about the dynamic control of the uterus than we do about the digestive tract. At a gross level, in healthy individuals the uterus shows cyclic peristaltic waves oriented downwards and outwards at menses with the isthmus, connecting the uterus to the oviduct, closed to ensure the correct expulsion of menstrual fluid. At mid-cycle ovulation, when the organism is sexually most receptive, the directionality of peristalsis is reversed, with contractility at ejaculation aimed at conveying a bolus of ejaculated semen to the upper end of the uterus and, depending upon the side of the ovulating ovary, with the ipsilateral opening of the isthmus to allow sperm to reach the site of fertilization in the ipsilateral oviduct (Kunz and Leyendecker, 2002). During implantation and pregnancy the myometrium becomes more or less quiescent, following a period of regulated mild contraction to assist implantation and embryo spacing (particularly in polytocous species). This changes dramatically at term, when oxytocin-induced contractility leads to the expulsion of the fetus and placenta and subsequent involution of the uterus.

This is the ideal scenario for most mammals, though in humans it appears that there may be severe disruption of one or other aspect. For example, it is now considered that endometriosis may have its origin in a retrograde peristalsis at menses, whereby menstrual material with resident stem cells is transported antero-dorsally through an open isthmus to enter the peritoneal cavity. This can occur in women at any time after puberty. Secondly, it has been shown for women attending an infertility clinic, but otherwise with positive reproductive parameters, that the direction of uterine peristalsis, instead of being toward an open ipsilateral isthmus at mid-cycle, is rather in the opposing direction, or that the ipsilateral isthmus is closed (Kunz and Leyendecker, 2002). Routine reproductive diagnosis generally does not assess uterine dynamics, largely one suspects because there is very little therapeutically that can be done. The research in this area is very sporadic and several decades old, in spite of the obvious translational implications. We still have no idea about the causes of uterine peristaltic directionality, or the way in which cycle-dependent hormones may modify this. Early attempts to assess the influence of steroids and oxytocin were without significant result (Kunz et al., 1998). In the gut, such directionality is co-regulated by resident Cajal-like cells; there is still little information at all about the presence or function of similar cells (also called telocytes) in the uterus of any species (Hutchings et al., 2009; Dodds et al., 2015). There is a very obvious need here for intensive research into the basic biology of the system and about appropriate methods of early diagnosis and treatment.

## Inaccessibility of the mid-gestation fetus and DOHaD

A major issue in understanding early fetal development and the progression of pregnancy is that the key period of fetal organogenesis, as well as of placental establishment and growth is also a period when the uterine contents are least accessible to analysis. Other than high resolution ultrasound and associated Doppler blood-flow measurements, the growing fetus and placenta are effectively invisible to the researcher. Some progress has been made by looking at fetal cells isolated from the maternal bloodstream, particularly as a source of fetal DNA. One very useful instrument had been the analysis of amniotic fluid collected at routine amniocentesis, i.e., approximately between weeks 12 and 20 of human pregnancy (Jensen et al., 2015). However, the risks involved for mother and child, and the development of alternative sources of fetal DNA (see above), have led to a significant reduction in such analyses, and anyway amniocentesis was always restricted to a limited number of indicated cases only. There is a definite need for other novel surrogates of fetal and placental well-being, particularly during the important transition from first to second trimester, when the risk of miscarriage is also high.

Another reason why this is so important is that this is a time when maternal and exogenous impacts on placental and fetal development are likely to have a considerable long-term effect on the health of the offspring. The Developmental Origins of Health and Disease (DOHaD) hypothesis, which is well-founded using animal models, as well as through epidemiological studies (Hanson and Gluckman, 2014), makes it clear that subtle influences (such as maternal obesity or diabetes) on fetal organogenesis and physiology can have life-long consequences. Monitoring of such early effects (preferably using biochemical surrogates) could go a long way toward developing appropriate timely interventional strategies and defining the molecular basis of such impacts.

## Sperm as non-somatic cells

The male gamete is a highly unusual cell. It is the only cell type to have its principal function outside of the body; it is also largely unable to transcribe or repair its DNA. In its mature form it has extremely little cytoplasm, effectively prohibiting diffusion kinetics from playing a major role in cell signaling and physiology. Instead the plasma membrane and other intracellular membrane structures host most of the functional dynamism required for the cell to fulfill its function, with the plasma membrane exhibiting a marked and changing spatial variability during the transit of the sperm from epididymal storage to oocyte fertilization. It is also enclosed by a dense and highly variable glycocalyx, which at ca. 200 μm certainly impacts on any inter-cellular/inter-molecular interactions. We now know that once sperm are released from quiescence during storage in the cauda epididymis and following the temperature and pH shock at ejaculation upon being released from scrotal coolness (32°C) to the relative warmth (37°C) of the female tract, they are already embarked on an apoptotic pathway (Koppers et al., 2011). Only those gametes which proceed to the oviduct and fertilize an oocyte can escape this fate. All the remainder undergo apoptotic cell death and are phagocytosed. This image of the spermatozoon is thus very different from that of any conventional somatic cell, and yet we still apply many of the paradigms derived from researching effectively immortal somatic cells when we try to understand sperm function. Future research must work within a more holistic sperm-centric environment if we are to understand better the physiology and pathology of the spermatozoon.

## Gonadal programming of puberty and old age

Age-related debility and disease is one of the greatest socio-economic burdens on global society, yet we still know relatively little about its biological etiology and consequently have little to offer in terms of prevention. The function of the ovaries and testes in this context is not well-understood. For women, the loss of oocyte recruitment at the menopause and resulting rapid decline in follicle-derived sex hormones is well-established and the basis for a steroid-based hormone-replacement therapy (HRT). But the sex steroids are not the only ovarian products to decline with age. The follicle-derived insulin-like peptide 3 (INSL3), which is essential to promote steroid production during reproductive life (Glister et al., 2013), also declines, as also anti-mullerian hormone and inhibin. For INSL3, there is evidence to suggest that this may have a direct impact on bone cells to promote bone formation and limit osteoporosis (Ferlin et al., 2013). Even for the sex steroids we still have only a poor understanding of the possible roles of their metabolites. Thus, there is still much room for a more comprehensive understanding of aging in women and possible interventional options. We also have only a limited understanding of the relationship between pre-menopausal reproductive status and endocrinology and post-menopausal sequelae. Nor do we have much understanding of what determines when menopause sets in, nor how many oocytes are contained within the young ovary and their ability to form follicles, both of which will determine sex hormone output during reproductive life.

For men, an age-related decline in testosterone production is associated with reduced cognitive ability, cardiovascular disease, reduced muscle, and bone strength, besides sexual capacity. Yet recent research has shown that simply replacing testosterone does not necessarily ameliorate these symptoms. Firstly, the Leydig cells of the testes which produce almost all of the testosterone in the body also generate large amounts of circulating INSL3 as well as 25-OH vitamin D with impacts on bone metabolism (Ferlin et al., 2013). Secondly, using INSL3 as a robust surrogate for the testes' capacity to produce testosterone suggests that this functional capacity is already established in young men, with life-long consequences for their ability to produce androgens in old age. Thus, because the Leydig cells appear neither to divide nor to regenerate across the adult lifespan, it seems for men that what they receive after puberty is likely to influence their capacity to make testosterone in later life. Yet we have very little understanding of what determines testis growth, development, and final size across puberty for any species.

Finally, well-being in adult life is very much a reflection of gonadal function. When this declines in old age, finding an appropriate replacement will be key to determining whether an ever extended lifespan will be worthwhile.

## EDCs and their impacts

Environmental EDCs are pervasive substances found in a wide range of consumer products, cosmetics, urban pollutants, and biocides. An EDC is defined as “*an exogenous substance or mixture that alters function(s) of the endocrine system and consequently causes adverse health effects in an intact organism, or its progeny, or (sub) populations*” (WHO/IPCS, 2002). Evidence is accumulating that EDCs may be impacting negatively on human as well as wildlife populations, and with consequences particularly for reproductive parameters, though other physiological systems are also shown to be affected (Gore et al., 2015). EDC science is controversial, not only because of the large industrial and political interests involved, but also because of the nature of the evidence which is weighed. EDCs are generally present in the environment and in body fluids in very low concentration. Robust epidemiological studies in large human populations suggest significant associations between EDCs and reproductive parameters (e.g., cryptorchidism, reduced anogenital distance) at concentrations well below levels considered safe in conventional toxicology studies. The problem is that animal studies at such low EDC concentrations would require prohibitively large numbers of animals to have sufficient power to detect the level of symptom penetrance seen in human or wildlife populations. Moreover, other than interfering with endogenous endocrine systems, we still have only a poor understanding of the molecular mechanisms involved, thus effectively proscribing the development of suitable *in vitro* assays. One reason for this may be that EDCs act by disrupting or perturbing the kind of signaling networks discussed previously above, and that impacts may be manifest only as significant changes to parameter variance rather than to the mean. Another reason is that EDCs are impacting particularly during development, for example during fetal organogenesis in the early second trimester, when a very small impact on a highly dynamic and differentiating cellular system can have large downstream effects on cell number, differentiation status, and/or epigenetics. The subtlety of such impacts is evidenced by the observation of significant changes to reproductive parameters only in the subsequent generations from the exposed individuals, pointing to long-lasting epigenetic changes in the germline. Though such effects may be small, the socio-economic consequences can be huge with estimates only for reproductive impacts in the order of several billion Euros for Europe or the USA every year (Hauser et al., 2015; Attina et al., 2016). There is a major research need here to develop sensitive systems which can satisfactorily predict EDC activity, and which can be applied for regulatory purposes, though as outlined above, this may require some considerable paradigm shifts away from conventional testing strategies.

Although this section has focused on EDCs, much of the subject area is also relevant for other exogenous interventions, including nutritional and lifestyle factors, as well as those inducing oxidative stress.

## The consequences of artificial selection

Domestic livestock and companion species have been subjected to targeted trait selection for many hundreds of years. In many instances this targeted selection for one characteristic has led to a concomitant worsening of reproductive parameters. An obvious example is the relative infertility of high-performance dairy cattle (Walsh et al., 2011), where evidently high milk yield is leading to significant negative energy balance at the time of implantation and gestation, though other factors may also be involved. Besides shifting to a more weighted multi-trait breeding program, what is required here are improved methods of *in vitro* oocyte maturation and embryo production, with appropriately reliable biomarkers, improved determination of estrus, and a better understanding of the substantial early pregnancy loss. That ~60% of pregnancy attempts are failing for one reason or another represents a huge economic burden for the dairy industry. What is notable is that there appears to be little improvement in fertility statistics over the last decade, in spite of substantial research input (Sheldon and Dobson, 2003). In pigs, selection for high fecundity has also led to increased fetal morbidity and runting (Vallet et al., 2013), showing that litter size alone is not a sufficient parameter, and that we need a better understanding of the relationship between number of fetuses and their health outcome.

However, the livestock industry is also undergoing a paradigm shift, of which we need to take account. In a more ethically conscious world, increasing hormonal or antibiotic interventions is no longer desirable. We need to develop alternative routes (nutritional, behavioral, or via improved management) to achieve similar effects.

## Concluding remarks

Research in reproduction has progressed considerably in recent decades, particularly in regard to advances in IVF procedures and reproductive biotechnology. These in turn have opened up new opportunities not only for human reproduction, but also for livestock species as well as endangered wildlife. There is still much potential for further developments in these areas, particularly in overcoming current technical limitations, for example, in assisted reproductive techniques for endangered species, or in regard to male germline transgenesis. The topics listed above are not intended to be comprehensive, but more to illustrate the kinds of new thinking in basic research that will be required to make significant advances in our field. There has also been little mention here of the major diseases of the reproductive system, such as polycystic ovarian syndrome, endometriosis, or cancers of the endometrium, breast, ovary, testis, and prostate. These are all extremely important areas of research endeavor, whose understanding will also require similar shifts in our thinking.

The reproductive system is a very special organ system which is responsible not only for producing gametes and sex hormones, but also because it is fundamental to our concept of individual well-being. Its failures may not be life-threatening, but its health is key to a robust old-age, to a rich biodiversity, and to considerable economic advantage.

This new Frontiers section on “Reproduction” aims to present to its readers new areas of research which represent an evolution in our thinking about the basic physiology of reproduction, as well as its translation into clinical, environmental and livestock practice. We welcome not only original research articles and reviews, but also hypotheses and novel concepts, so that our field can be stimulated to grow and excel.

## Author contributions

RI, conceived and wrote the entire manuscript. All opinions expressed therein are his alone.

### Conflict of interest statement

The author declares that the research was conducted in the absence of any commercial or financial relationships that could be construed as a potential conflict of interest.
